# Serum human trophoblast cell surface antigen and T-box protein 5 levels play a role in predicting adverse pregnancy outcomes in eclampsia

**DOI:** 10.5937/jomb0-60246

**Published:** 2026-04-15

**Authors:** Yao Li, Sutong Huang, Yan Xuan, Hao Shen, Weiwei Zhang, Jia Li

**Affiliations:** 1 Aoyang Hospital Affiliated to Jiangsu University, Department of Obstetrics and Gynaecology, Zhangjiagang City, Jiangsu Province, China; 2 Huai'an Clinical Medical College of Jiangsu University (Huai'an Hospital of Huai'an City), Department of Obstetrics and Gynaecology, Huai'an City, Jiangsu Province, China; 3 Rongcheng People's Hospital of Shandong Province, Psychological Consulting Room, Rongcheng City, Shandong Province, China; 4 Digestive Disease Institute of Jiangsu University, Department of Gastroenterology, Jiangsu University Affiliated Hospital, Zhenjiang, Jiangsu Province, China; 5 Huai'an Hongze District People's Hospital, Department of Obstetrics and Gynaecology, Huai'an City, Jiangsu Province, China

**Keywords:** serum human trophoblast cell surface antigen, T-box protein 5, adverse pregnancy, eclampsia, serumski humani trofoblastni površinski antigen, T-box protein 5, nepovoljni ishod trudnoće, eklampsija

## Abstract

**Background:**

The levels of human trophoblast surface antigen (Trop-2) and T-box protein 5 (TBX5) in the serum of patients with preeclampsia were detected, and their value in predicting adverse pregnancy outcomes was analysed.

**Methods:**

A total of 196 pregnant women with preeclampsia who underwent prenatal examinations and delivered in our hospital from October 2021 to September 2024, as well as 196 healthy pregnant women, were selected as the eclampsia group and the control group, respectively. The 196 pregnant women with preeclampsia were divided into two groups according to their pregnancy outcome: the adverse outcome group and the good outcome group. Using the enzyme-linked immunosorbent assay (ELISA), the levels of serum Trop-2 and TBX5 were measured. Using multivariate logistic regression analysis, the factors impacting the various pregnancy outcomes of preeclamptic pregnant women were examined.

**Results:**

Compared with the control group, the serum TBX5 levels in the eclampsia group (2 0.8 6± 6 .4 5 mg/L vs. 15.34± 5.07 mg/L), as well as diastolic blood pressure, FBG, 24-hour urine protein, TG, systolic blood pressure and TC, were significantly greater. The levels of Trop-2 ((5.46± 1 .7 2) mg/L vs. (7 .37 ± 2.08 ) mg/L). The total incidences of preterm infants, fetal distress, placental abruption and adverse pregnancy outcomes in the eclampsia group were significantly greater than those in the control group (P&lt; 0.05). The serum TBX5 level in the adverse outcome group ((23.01± 6.66) mg/L vs. (1 9.53± 6.32) mg/L), as well as diastolic blood pressure, FBG, 24-hour urine protein, TG, systolic blood pressure, and TC, were significantly greater than those in the good outcome group. The levels of Trop-2 ((4.90± 1.61) mg/L vs. (5.81± 1.79) mg/L) and HDL-C decreased significantly (P&lt; 0.05); Trop-2 (OR 95% CI: 0.38 (0.28, 0.53)), TBX5 (OR 95% CI: 3.89 (1.88, 8.04)) and 24-hour urine protein (OR: 3.14 (1.82, 5.42)) were influencing factors for the pregnancy outcome of pregnant women with preeclampsia (P&lt; 0.05); and the AUCs of serum Trop-2, TBX5 and their combination in predicting the pregnancy outcome of pregnant women with preeclampsia were 0.79, 0.76 and 0.90, respectively. The combined prediction of the two had a greater predictive value than did Trop-2 or TBX5 alone (all P&lt;0.05).

**Conclusions:**

The serum Trop-2 level of pregnant women with preeclampsia is abnormally low, and the TBX5 level is abnormally high. The combination of the two can be used as a biological indicator for evaluating adverse pregnancy outcomes.

## Abstract (sr)

**Uvod:**

Cilj je bio da se ispitaju nivoi humanog trofoblastnog površinskog antigena (Trop-2) i T-box proteina 5 (TBX5) u serumu pacijentkinja sa preeklampsijom i analizirati njihov značaj u predviđanju nepovoljnih ishoda trudnoce.

**Metode:**

U istraživanje je uključeno ukupno 196 trudnica sa preeklampsijom, koje su obavile prenatalne preglede i porodile se u našoj bolnici u periodu od oktobra 2021. do septembra 2024. godine, kao i 196 zdravih trudnica. One su raspoređene u grupu sa preeklampsijom i kontrolnu grupu. Trudnice sa preeklampsijom dalje su podeljene na grupu sa nepovoljnim i grupu sa povoljnim ishodima, u zavisnosti od toka trudnoce. Nivoi Trop-2 i TBX5 u serumu određivani su metodom enzimski povezanog imunosorbentnog testa (ELISA). Multivarijantnom logističkom regresionom analizom ispitivani su faktori koji utiču na ishod trudnoce kod trudnica sa preeklampsijom.

**Rezultati:**

U poređenju sa kontrolnom grupom, kod grupe sa preeklampsijom utvrđeni su značajno viši nivoi TBX5 u serumu (2 0,8 6± 6 ,4 5 mg/L naspram 15,34± 5,07 mg/L), kao i viši dijastolni krvni pritisak, glikemija natašte (FBG), 24-časovna proteinurija, trigliceridi (TG), sistolni krvni pritisak i ukupni holesterol (TC), dok su nivoi Trop-2 bili značajno niži (5,46± 1,72 mg/L naspram 7,37± 2,08 mg/L). Ukupna učestalost prevremenog porođaja, fetalnog distresa, abrupcije placente i nepovoljnih ishoda trudnoće bila je značajno viša u grupi sa preeklampsijom (P&lt; 0,05). Unutar grupe sa preeklampsijom, kod trudnica sa nepovoljnim ishodima utvrđeni su viši nivoi TBX5 (23,01 ±6,66 mg/L naspram 19,53± 6,32 mg/L), kao i viši dijastolni krvni pritisak, glikemija nataste, 24-časovna proteinurija, trigliceridi, sistolni krvni pritisak i ukupni holesterol, u poređenju sa grupom sa povoljnim ishodima. Nivoi Trop-2 (4,90± 1,61 mg/L naspram 5,81± 1,79 mg/L) i HDL-C bili su značajno niži (P&lt; 0,05). Analizom je utvrđeno da su Trop-2 (OR 95% CI: 0,38 (0,28-0,53)), TBX5 (OR 95% CI: 3,89 (1,88-8,04)) i 24-časovna proteinurija (OR: 3,14 (1,82-5,42)) faktori koji utiču na ishode trudnoće kod pacijentkinja sa preeklampsijom (P&lt; 0,05). AUC vrednosti Trop-2, TBX5 i njihove kombinacije u predviđanju ishoda trudnoće kod žena sa preeklampsijom bile su 0,79, 0,76 i 0,90. Kombinacija Trop-2 i TBX5 imala je veću prediktivnu vrednost nego svaki marker pojedinačno (svi P&lt; 0,05).

**Zaključak:**

Kod trudnica sa preeklampsijom nivo Trop-2 u serumu je značajno snižen, dok je nivo TBX5 značajno povišen. Njihova kombinacija može poslužiti kao biološki marker za procenu rizika od nepovoljnih ishoda trudnoće.

## Introduction

Preeclampsia is a type of idiopathic hypertension during pregnancy, usually referred to as normal blood pressure before conception. Still, symptoms such as proteinuria, elevated blood pressure, dizziness and headache occur after 20 weeks of pregnancy. The incidence of preeclampsia in pregnant women is estimated to be approximately 5% [1]. Preeclampsia can progress to eclampsia, causing convulsions or coma in patients and even leading to serious maternal and fetal complications, including severe complications of gestational hypertension such as HELLP syndrome, cardiovascular and cerebrovascular diseases, renal failure, liver injury, fetal growth restriction, fetal nervous system damage, fetal distress, oligohydramnios, and fetal death [2][3].

Therefore, timely control of preeclampsia and intervention in its possible consequences are significant for improving the adverse pregnancy outcomes of pregnant and postpartum women [4]. Studies have shown that during pregnancy, maternal Trop-2 is regulated by miRNA (miR)-125b and is indirectly involved in placental development [5]. The epicardium, myocardium, endocardium, and other tissues of both embryonic and adult hearts display the T-box domain, which is found in T-box protein 5 (TBX5), a member of the T-box transcription factor family [6]. Studies have shown that TBX5 can cause fetal arrhythmia and is related to the development process and/or functional status of the cardiac conduction system [7][8]. However, clinical studies of Trop-2 and TBX5 in preeclampsia have not yet been reported.

Therefore, in this study, the levels of Trop-2 and TBX5 in the serum of pregnant women with preeclampsia were detected to analyse their predictive value for adverse pregnancy outcomes.

## Materials and methods

### General information collection

A total of 196 pregnant women with preeclampsia who underwent prenatal examinations and gave birth at our hospital from October 2021 to September 2024 composed the eclampsia group, and another 196 healthy pregnant women who underwent prenatal examinations and gave birth at the same hospital during the same period composed the control group. There was no statistically significant difference in age, parity, or gestational age at enrollment.

### Inclusion criteria

(1) Met the relevant diagnostic criteria for preeclampsia and had a singleton pregnancy and natural conception; (2) The gestational age at enrollment was 24-35 weeks. There was no history of hypertension before pregnancy and no bad habits, such as excessive drinking, staying up late, or smoking; (3) The clinical data of the pregnant woman were complete.

### Exclusion criteria

Complicated with major diseases of the heart, kidneys, liver, etc.; (2) Combined with other special diseases during pregnancy or accompanied by malignant tumours or other factors that may affect the results of this study; (3) Combined with coagulation dysfunction and immune system diseases; (4) Concurrent mental or psychological disorders.

### Collation of clinical data

The systolic and diastolic blood pressures of pregnant women during the same period were detected via an automatic pulse wave blood pressure monitor (Jinan Laibao Medical Devices Co., Ltd.). The total urine volume of the enrolled pregnant women from 7 a.m. on the first day (with the bladder emptied at 7 a.m.) to 7 a.m. on the second day was collected. The urine was mixed evenly, and 20-50 ml was collected. The urine protein concentration was determined via a Cybow720 urine analyser from Cybo, South Korea (Shanghai Hanfei Medical Devices Co., Ltd.), and the 24-hour urine protein volume was calculated based on the total urine volume.

### Levels of Trop-2 and TBX5

The serum was separated via centrifugation at 1789xg for 10 minutes using a German Ebende Centrifuge 5910 Ri refrigerated centrifuge (Kezhan Biotechnology Co., Ltd.). The levels of serum Trop-2 and TBX5 were detected by using a Thermo Fisher Varioskan LUX multifunctional microplate reader (Qingdao Jiading Analytical Instrument Co., Ltd.). The levels of Trop-2 and TBX5 in the serum of the pregnant women in the group were calculated based on the standard curve.

### Grouping of pregnancy outcomes

The eclampsia group and the control group were statistically compared for the incidence of poor pregnancy outcomes, including maternal heart failure, postpartum haemorrhage, newborn asphyxia, placental abruption, fetal distress, fetal mortality, and premature birth. Pregnant women with the above adverse pregnancy outcomes in the eclampsia group were classified into the adverse outcome group. Pregnant women in the eclampsia group who did not have the above conditions were classified into the good outcome group, with a total of 121 cases.

### Statistical methods

The obtained data were described and analysed via SPSS 25.0 statistical software. Two independent sample t-tests were used for comparisons between two groups. The counting data are expressed as examples (%), and the 2 test was used for comparison. The predictive value of serum Trop-2 and TBX5 levels for different pregnancy outcomes in pregnant women with preeclampsia was analysed via receiver operating characteristic (ROC) curves, and the area under the curve (AUC) was compared via the Z test. P<0.05 indicated a statistically significant difference.

## Results

### Comparison of serum Trop-2 and TBX5 levels between the eclampsia group and the control group

The serum Trop-2 level in the eclampsia group was significantly lower than that in the control group (5.46±1.72 μg/L vs. 7.37±2.08 μg/L, t=9.91, P<0.001), and the TBX5 level was significantly greater than that in the control group (20.86±6.45 μg/L vs. 15.34±5.07 μg/L, t=9).42, P<0.001) (Table 1).

**Table 1:** Comparison of general data between healthy pregnant women (control group) and pregnant women with preeclampsia (eclampsia group).

Group	Number of <br>cases	Age <br>(years, x̄±s)	First labour/<br>first labour/case	Gestational age at enrollment<br>(Week, x̄±s)
Control group	196	29.43±3.69	120/76	30.25±3.61
Eclampsia group	196	28.85±3.72	105/91	29.67±3.48
t(x^2^) value		1.55	(2.35)	1.62
P value		0.122	0.125	0.106

### Comparison of clinical data between the eclampsia group and the control group

As shown in Table 2, the eclampsia group's diastolic blood pressure, systolic blood pressure, FBG, TG, TC, and 24-hour urine protein were significantly greater (P<0.05) than those of the control group, whereas HDL-C was significantly lower (P<0.05).

**Table 2:** Comparison of clinical data between healthy pregnant women (control group) and pregnant women with preeclampsia (eclampsia group) /x̄±s.

Group	Number<br>of cases	Diastolic blood<br>pressure<br>(mmHg)	Systolic blood<br>pressure<br>(mmHg)	FBG <br>(mmol/L)	TG <br>(mmol/L)	TC <br>(mmol/L)	HDL-C <br>(mmol/L)	24-hour urine<br>protein<br>(g/24h)
Control <br>group	196	82.34±8.07	125.49±10.63	4.30±1.15	2.02±0.64	4.75±1.53	1.20±0.36	0.21±0.07
Eclampsia <br>group	196	104.51±9.62	156.38±14.16	7.46±2.08	3.11±0.97	5.26±1.61	0.85±0.24	1.58±0.46
t value		24.72	24.42	18.61	13.13	3.22	11.33	41.22
P value		<0.001	<0.001	<0.001	<0.001	0.001	<0.001	<0.001

### An analysis comparing the prevalence of unfavourable pregnancy outcomes

The incidence of fetal death in the two groups did not differ statistically significantly (P>0.05). Table 3 indicates that the eclampsia group had significantly higher overall incidence rates of fetal distress, placental abruption, preterm newborns, and unfavourable pregnancy outcomes (P<0.05) than the control group.

**Table 3:** Comparison of the incidence of adverse pregnancy outcomes between healthy pregnant women (control group) and pregnant women with preeclampsia (eclampsia group)/case (%).

Group	Number<br>of cases	Premature <br>infants	Fetal <br>distress	Fetal <br>death	Placental <br>abruption	Neonatal <br>asphyxia	Postpartum <br>hemorrhage	Maternal<br>heart failure	Always <br>happen
Control <br>group	196	23(11.73)	14(7.14)	0(0)	0(0)	2(1.02)	7(3.57)	0(0)	36(18.37)
Eclampsia <br>group	196	61(31.12)	44(22.45)	2(1.02)	28(14.29)	9(4.59)	57(29.08)	7(3.57)	90(45.92)
x^2^ value		21.88	18.21	0.50	30.15	4.58	46.68	5.24	34.11
P value		<0.001	<0.001	0.478	<0.001	0.032	<0.001	0.022	<0.001

### Clinical information and serum Trop-2 and TBX5 levels of preeclamptic pregnant women with varying pregnancy outcomes are compared.

As indicated in Table 4, the adverse outcome group's serum TBX5 level, diastolic blood pressure, FBG, systolic blood pressure, TG, 24-hour urine protein, and TC were significantly higher (P<0.05) than those in the good outcome group, while the Trop-2 level and HDL-C were significantly lower (P<0.05).

**Table 4:** Comparison of serum Trop-2 and TBX5 levels and clinical data of pregnant women with preeclampsia with different pregnancy outcomes /x̄±s.

Group	Number<br>of cases	Trop-2 <br>(μg/L)	TBX5 <br>(μg/L)	Diastolic blood<br>pressure<br>mmHg	Systolic blood<br>pressure<br>mmHg
Good Outcome Group	121	5.81±1.79	19.53±6.32	99.16±9.47	151.54±13.96
Adverse Outcome<br>group	75	4.90±1.61	23.01±6.66	113.14±9.86	164.19±14.48
t value		3.59	3.67	9.89	6.08
P value		<0.001	<0.001	<0.001	<0.001
Group	FBG <br>(mmol/L)	TG <br>(mmol/L)	TC <br>(mmol/L)	HDL-C <br>(mmol/L)	24-hour urine<br>protein<br>(g/24h)
Good Outcome Group	6.78±2.05	2.98±0.94	5.01±1.56	0.89±0.23	1.51±0.47
Adverse Outcome<br>group	8.56±2.13	3.32±1.02	5.66±1.69	0.79±0.26	1.69±0.44
t value	5.82	2.38	2.75	2.81	2.67
P value	<0.001	0.018	0.007	0.005	0.008

### Analysis of various pregnancy outcomes using multivariate logistic regression in pregnant patients with preeclampsia

The pregnancy outcome of pregnant women with preeclampsia was used as the dependent variable (adverse outcome =1, good outcome =0), and Trop-2, TBX5, diastolic blood pressure, FBG, systolic blood pressure, TG, 24-hour urine protein, total cholesterol (TC) and high-density lipoprotein cholesterol (HDL-C) (continuous variables) were used as independent variables. The above variables were input into the multiple logistic regression analysis. A regression model was established via the forward method. The results revealed that Trop-2, TBX5 and 24-hour urine protein were the influencing factors of pregnancy outcomes in pregnant women with preeclampsia (P<0.05) (Table 5).

**Table 5:** Multivariate logistic Regression analysis of different pregnancy outcomes in 196 pregnant women with preeclampsia.

Item	β value	Standard error	Wald x^2^ value	OR value	95%CI	P value
Trop-2	-0.96	0.16	34.91	0.38	(0.28, 0.53)	<0.001
TBX5	1.36	0.37	13.39	3.89	(1.88, 8.04)	<0.001
24-hour urine protein	1.15	0.28	16.95	3.14	(1.82, 5.42)	<0.001

### Predictive value of serum Trop-2 and TBX5 for the pregnancy outcome of pregnant women with preeclampsia

The ROC curves of serum Trop-2 and TBX5 for predicting the pregnancy outcome of pregnant women with preeclampsia are shown in Table 6. The combined prediction of serum Trop-2 and TBX5 had a greater predictive value than did Trop-2 (Z = 2.88, P=0.002) or TBX5 (Z=3.29, P=0.001) alone.

**Table 6:** The predictive value of serum Trop-2 and TBX5 for the pregnancy outcomes of 196 pregnant women with preeclampsia.

Indicator	AUC	95%CI	Truncated value (μg/L)	Sensitivity 1%	Specificity 1%
Trop-2	0.79	(0.73, 0.85)	5.33	78.70	73.60
TBX5	0.76	(0.70, 0.83)	20.88	80.00	67.80
Combination of the two	0.90	(0.86, 0.94)		72.00	

## Discussion

Preeclampsia is a multisystem, placenta-mediated hypertensive disorder characterised by systemic inflammation, oxidative stress, endothelial dysfunction and vascular injury [8]. Owing to the extremely complex composition of the maternal fetal interface, the aetiology of preeclampsia is not yet fully clear. The fundamental cause of preeclampsia is generally believed to be placental ischemia. However, its occurrence is multifactorial and highly heterogeneous [9]. Furthermore, the ability of existing markers to predict pregnancy outcomes in patients with preeclampsia is limited [10]. Therefore, identifying timely and effective biomarkers is highly important for predicting the pregnancy outcome of pregnant women with preeclampsia.

In a normal pregnancy, appropriate placenta accreta is of vital importance. The extravillous trophoblast invades the maternal endometrium and myometrium, providing abundant maternal blood to the villous spaces of the placenta and maintaining fetal growth [11]. However, in the early stage of pregnancy with preeclampsia, invasion of extravillous trophoblasts is disrupted, placental perfusion is reduced, and neovascularisation is disrupted. The remodelling of the maternal vessels supplying the villous spaces is insufficient to maintain a normal pregnancy [12]. Reduced placental perfusion and vascular endothelial injury lead to an increase in various cytokines in the trophoblast and placenta. These cytokines eventually cause preeclampsia by causing endothelial cell injury and dysfunction, and simultaneously result in fetal growth restriction, placental abruption, and spontaneous preterm birth [13]. In addition, cytokines can stimulate the sensitivity of blood vessels to pressure and vascular permeability, thereby damaging multiple organs of the mother, including the liver, kidneys, brain, heart, and eyes. Both early-onset and late-onset preeclampsia are associated with trophoblasts [14]. Trop-2 is a transmembrane glycoprotein of 35-49 kDa that is involved in regulating intercellular adhesion and cell proliferation and maintaining the integrity of the basement membrane.

Furthermore, Trop-2 is considered a biomarker of germ cells and is related to the regeneration of various tissues. Trop-2 was initially discovered in placental trophoblasts, and cells expressing Trop-2 can invade the uterus during placental implantation [15]. In this study, the Trop-2 score in the eclampsia group was significantly lower than that in the control group, and the Trop-2 score in the adverse outcome group was lower than that in the good outcome group. These findings indicate that Trop-2 is involved in the occurrence of preeclampsia. The reason might be the elevated level of Trop-2, allowing embryonic trophoblast cells to invade the uterus normally. When the level of Trop-2 is low, the invasion ability of embryonic trophoblast cells is insufficient, resulting in inadequate placental perfusion and blocked angiogenesis. During this period, the cytokines produced cause damage to the fetus and the mother. As pregnancy continues, the remodelling of the maternal vessels supplying the villous space fails, and the damage caused by cytokines intensifies, eventually leading to preeclampsia.

In the early stage of cardiac development, TBX5 is activated and is related to cardiac morphological partitioning and the maturation of cardiomyocytes. In the later stage, it is also involved in the development of the cardiac conduction system [16]. Preeclampsia has an adverse effect on the plasticity of fetal development. The fetal cardiac remodelling pattern of pregnancy complicated with preeclampsia manifests as a larger heart, ventricular hypertrophy, an elevated myocardial function index, and elevated brain diuretic natriuretic peptide levels in umbilical cord blood [17][18]. In addition, abnormal cardiac remodelling during preeclampsia pregnancy is initiated or magnified, and the mother also shows increased sensitivity to angiotensin II and elevated levels of factors such as TBX5, thereby leading to an increased incidence of cardiovascular diseases [19][20][21][22][23], revealing that the TBX5 level is involved in the disease progression of preeclampsia.

Furthermore, this study showed that HDL-C in pregnant women with preeclampsia decreased significantly [24][25][26], whereas FBG, 24-hour urine protein, TG and TC levels increased significantly. This reveals that when preeclampsia occurs during pregnancy, abnormal changes also occur in blood glucose, blood lipid and 24-hour urine protein levels in the body. The total incidence of preterm infants, fetal distress, placental abruption and adverse pregnancy outcomes in the eclampsia group significantly increased [27]. The levels of TBX5 and 24-hour urinary protein increased. This study revealed that the combined value of the serum Trop-2 and TBX5 indicators for predicting the pregnancy outcome of pregnant women with preeclampsia was greater than that of Trop-2 and TBX5 alone, revealing that the combination of Trop-2 and TBX5 can be used as a better biological indicator for predicting the pregnancy outcome of pregnant women with preeclampsia [28].

In summary, the determinants of pregnancy outcome in pregnant women with preeclampsia include increased TBX5 levels and decreased serum Trop-2 levels. The combination of Trop-2 and TBX5 can be used as a biological indicator.

## Dodatak

### Authors' contribution

Yao Li and Sutong Huang contributed equally as first authors.

### Conflict of interest statement

All the authors declare that they have no conflict of interest in this work.
